# Atypical Presentation of Lemierre's Syndrome Causing Septic Shock and Acute Respiratory Distress Syndrome

**DOI:** 10.1155/2018/5469053

**Published:** 2018-07-02

**Authors:** Divyesh Reddy Nemakayala, Manoj P Rai, Shilpa Kavuturu, Supratik Rayamajhi

**Affiliations:** Department of Medicine, Michigan State University, 804 Service Road, Room B301, East Lansing, MI 48824, USA

## Abstract

Lemierre's disease is a rare but life-threatening condition characterized by an oropharyngeal infection complicating with thrombophlebitis of the internal jugular vein and disseminated abscesses. We are presenting a case of a young female who initially presented with fevers, chills, sore throat, and swollen neck later developed progressively worsening shortness of breath along with sudden onset pleuritic chest pain. She then developed progressively worsening acute hypoxic respiratory failure requiring intubation and mechanical ventilation. Interval chest X-ray showed worsening bilateral effusions. She also developed septic shock requiring pressors. Blood culture showed *Fusobacterium*, and antibiotics were changed accordingly following which there was a clinical improvement. The diagnosis of Lemierre's syndrome was then established based on her presenting age and bilateral pulmonary empyema in the setting of septicemia with *Fusobacterium*.

## 1. Introduction

Lemierre's syndrome typically begins with an oropharyngeal infection. It invades the pharyngeal mucosa to cause septic thrombophlebitis of the internal jugular vein (IJV). Thrombophlebitis of the IJV then seeds to cause bacteremia and metastatic septic emboli. *Fusobacterium* most commonly causes Lemierre's syndrome. Even though it was first reported by Courmont and Cade [1], a clear description was provided by André Lemierre in 1936 [1] and [2]. Here, we present an 18-year-old female who developed acute respiratory distress syndrome (ARDS) as a complication of Lemierre's syndrome.

## 2. Case

An 18-year-old female without significant past medical history initially presented to urgent care with complaints of a sore throat, swollen neck, fevers, and chills for 5 days. At the urgent care, the rapid strep test came back negative. She was then sent home on steroids and azithromycin. She presented to the emergency department two days later with progressively worsening shortness of breath along with sudden onset pleuritic chest pain. Review of systems was remarkable for shortness of breath and chest pain. Vitals showed temperature of 99 °F, blood pressure of 107/66 mm Hg, a pulse of 138/min, respiratory rate of 28/min, and SpO_2_ of 97%. Physical examination was remarkable for tenderness in the neck, pus formation on the tonsils, and decreased breath sounds. Labs were remarkable for severe thrombocytopenia, leukocytosis with left shift, granulated polymorphonuclear leukocytes (PMNs), and acute kidney injury (AKI).

**Figure 1:**
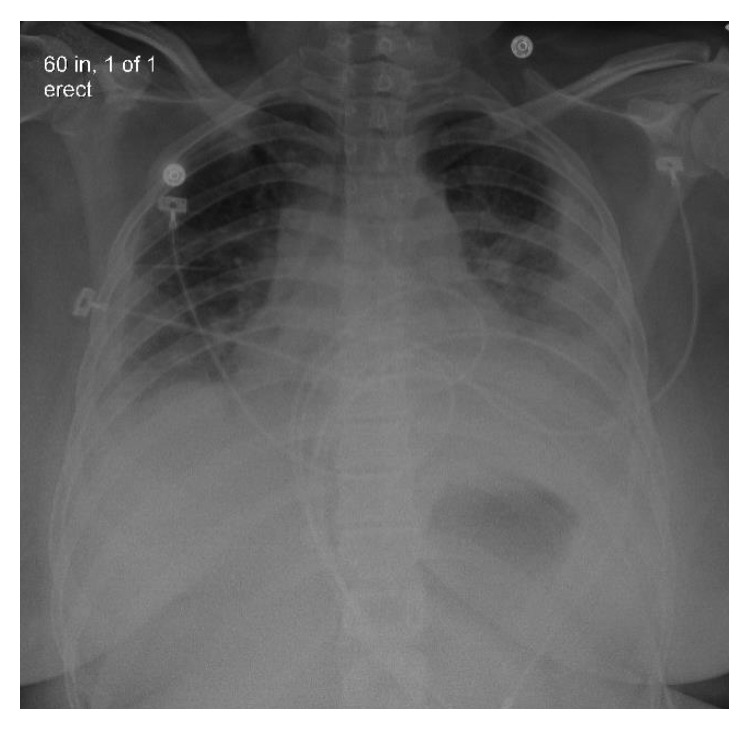
Chest X-ray showing bilateral pleural effusion.

**Figure 2:**
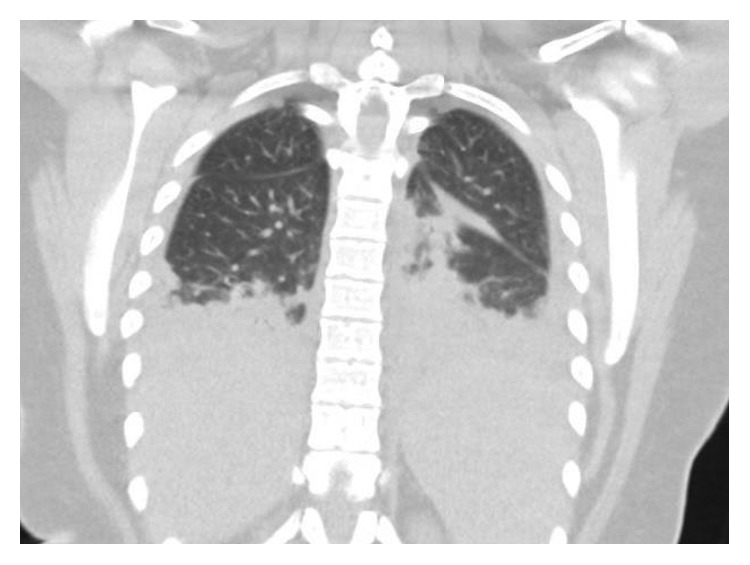
Computed tomography (CT) chest without contrast showed bilateral lung nodules and pleural effusions.

**Figure 3:**
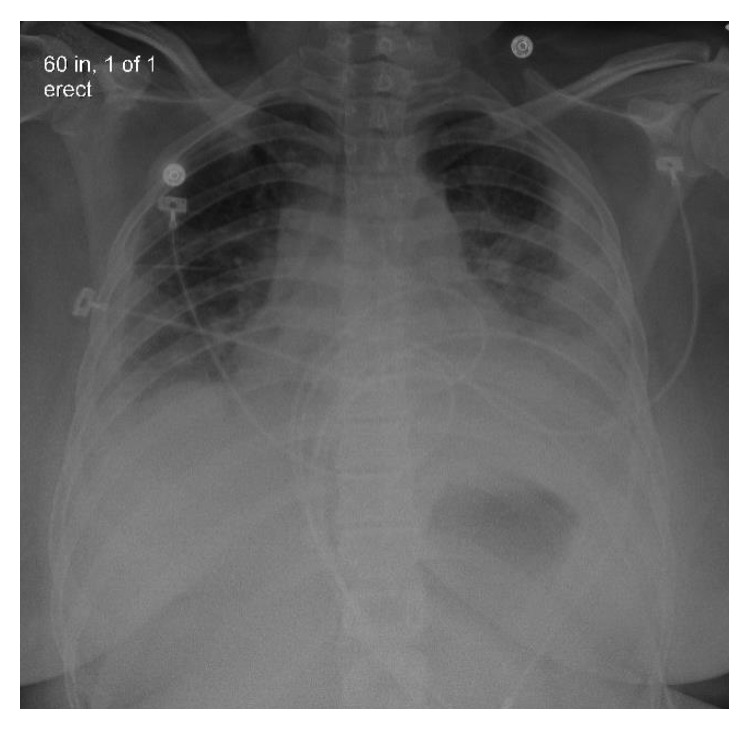
Interval chest X-ray showing worsening bilateral effusions.

**Figure 4:**
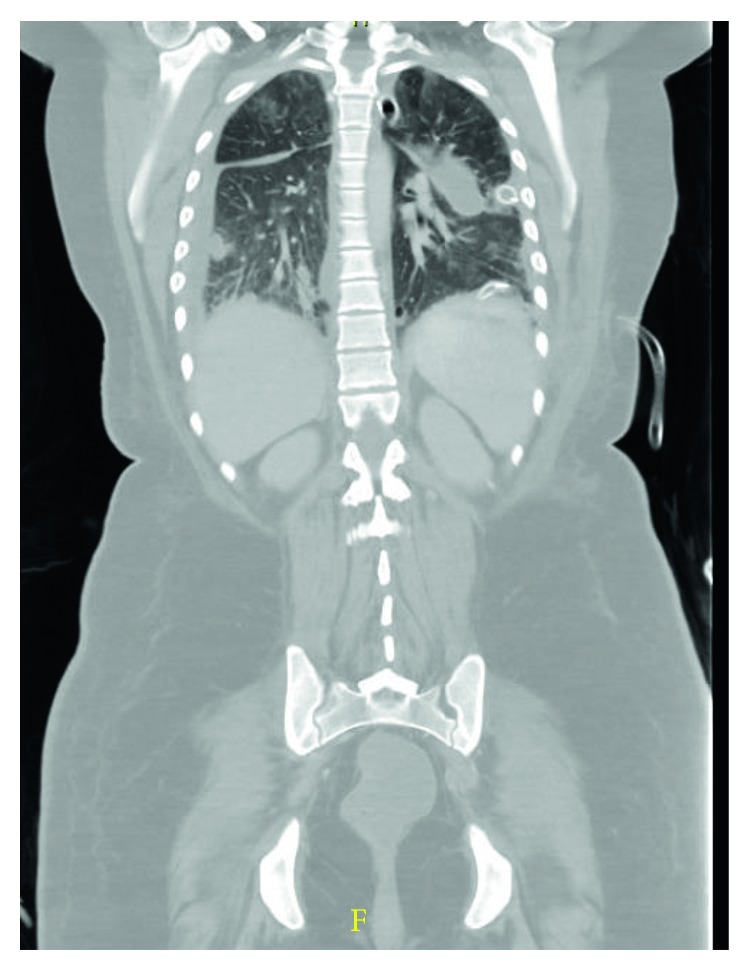
Repeat CT chest abdomen pelvis with contrast 5 days later showing septic emboli in the lungs bilaterally and bilateral loculated empyema.

Initial chest X-ray showed bilateral pleural effusions (Figure 1). Computed tomography (CT) chest without contrast showed bilateral lung nodules and pleural effusions (Figure 2). Echocardiogram demonstrated small pleural effusion with normal ejection fraction. Bilateral neck ultrasound and computed tomography (CT) neck without contrast did not show jugular vein thrombophlebitis or peritonsillar abscess, although the study was limited due to insertion of bilateral internal jugular (IJ) catheter insertions. Blood cultures were obtained, intravenous fluids were given, and empiric antibiotic therapy was started with intravenous (IV) vancomycin, IV cefepime, and IV doxycycline. The patient became more hypoxic requiring intubation and mechanical ventilation and went into septic shock requiring pressors. An interval chest X-ray demonstrated worsening bilateral effusions (Figure 3). Her renal function deteriorated requiring continuous renal replacement therapy (CRRT). She then developed cardiac arrest due to pulseless electrical activity (PEA) following chest compressions, and there was a return of spontaneous circulation (ROSC). Blood culture grew *Fusobacterium*, and antibiotics were changed to IV meropenem. To drain the empyema, bilateral chest tubes were placed, the sample was cultured, and it was negative for bacterial growth. The patient also had a left-sided pneumothorax, and it was unsure if the cause was secondary to the underlying infection or iatrogenic. She was placed on acute respiratory distress syndrome (ARDS) net protocol following which there was an improvement, and she was eventually weaned off the ventilator. A repeat CT chest abdomen pelvis with contrast 5 days later was ordered which showed septic emboli in the lungs bilaterally, splenic emboli, bilateral loculated empyema, and left adrenal gland hemorrhage (Figure 4).

### 2.1. Outcome and Follow-Up

The patient responded to antibiotics, and she required long-term care at LTACH where she recovered completely.

## 3. Discussion

This case illustrates an atypical presentation of oropharyngeal infection complicating to cause sepsis with multiorgan failure. Symptoms of a recurrent sore throat and neck pain symptoms in an otherwise healthy young adult should raise suspicion for Lemierre's syndrome. It will enable us to diagnose the condition at an early stage and start the patient on appropriate antibiotic treatment to reduce mortality. It appears that our patient had severe exudative pharyngitis which may have progressed to cause transient jugular venous thrombophlebitis manifested as neck swelling. Lemierre's syndrome is a rare disease. One of the prospective studies detected an annual incidence of 3.6 cases and an annual rate of 14.4 cases per million people among patients with age group from 14 to 24 years [3]. The commonly associated symptoms include swelling and pain in the neck. It is associated with signs of induration at the ipsilateral angle of the mandible of the neck extending along the sternocleidomastoid muscle along with high fevers, trismus, and bilateral or unilateral anterior cervical lymphadenopathy [1]. In cases with an infection involving posterior lateral pharyngeal space, the thrombosed internal jugular vein (IJV) may not be palpable [4]. Lemierre had described a triad of pleuritic chest pain, dyspnea, and hemoptysis and the presence of localized crackles and a pleural rub on auscultation. Pulmonary involvement is a common presentation of Lemierre's syndrome. It results from metastasis of septic emboli from the internal jugular vein through the pulmonary arteries. In 85% of patients, it manifests with pulmonary effusions, abscesses, and empyema. In about 10% of patients, it can complicate with pneumatoceles, pneumothorax, and acute respiratory distress syndrome [1]. It can cause septic arthritis, osteomyelitis, meningitis, pericarditis, and hepatic abscesses [5–7] which were absent in our patient. Along with cytokines, prostanoids, and nitric oxide, it has been postulated that coronary circulation and cardiomyocyte physiology also play major role in modulating the effects of monocyte adhesion and infiltration. Damage-associated molecular patterns (DAMPs) and pathogen-associated molecular patterns (PAMPs) are involved in the host response [8]. Lab workup typically shows a neutrophilic leukocytosis, thrombocytopenia, and an elevated C-reactive protein (CRP). Ultrasonography is often the first choice as it is a relatively cheap choice for visualization of the internal jugular vein [9]. It can miss newly formed thrombus with low echogenicity since it is less sensitive in the area deep to clavicle and mandible [10]. Contrast-enhanced CT of the neck has better specificity compared to the ultrasound in the detection of IJV thrombosis, and also it can detect other complications such as pulmonary emboli, abscesses, osteomyelitis, and arthritis [5, 11]. However, in our case, it could not be performed due to her reduced renal function. Magnetic resonance imaging (MRI) can also be used to rule out possible thrombi; however, it is expensive [2]. Detection and growth of a *Fusobacterium spp.* from anaerobic blood culture are required for the diagnosis of Lemierre's syndrome. It may sometimes take up to 7 days; however, in our case, blood culture was positive for *Fusobacterium* on day 1. The diagnosis of Lemierre's syndrome is established once the following criteria are met: primary anaerobic infection in the oropharynx, subsequent septicemia (with at least one positive blood culture), metastatic infection at one or more distant site, and thrombophlebitis of the IJV [12]. In our case, there was no evidence of thrombophlebitis of the IJV. However, the placement of the bilateral internal jugular vein catheters would have dislodged the thrombi, and the presence of catheters compromised the clear visualization of the veins. So, it could have been a false-negative result. Treatment involves the use of appropriate antibiotics and surgical drainage in some cases. Anticoagulation should be considered in some cases [1]. There are no specific guidelines to direct the antibiotic regimen in patients with Lemierre's syndrome. *Fusobacterium* is known to be resistant to *β*-lactams [13], so the addition of *β*-lactamase inhibitor should always be considered. We should also target oral streptococci.

The combination of ceftriaxone and metronidazole provides coverage for both *F. necrophorum* and oral streptococci. Metronidazole is the most commonly used antibiotic for *Fusobacterium* according to one of the case reports [14]. Data from one of the in vitro studies showed that metronidazole or imipenem has greater bactericidal activity against *F. necrophorum* than clindamycin [15]. Monotherapy with a carbapenem, ampicillin-sulbactam, antipseudomonal penicillins, or clindamycin is also appropriate antimicrobial options, except for carbapenem which cannot be used in patients who develop central nervous system (CNS) manifestations [16]. Antimicrobial resistance among *F. necrophorum* is rare; according to one of the studies, 15% were resistant to erythromycin and only 2% of strains were resistant to penicillin. Antimicrobial therapy should be given for 3 to 6 weeks [1, 2]. Management of septic shock with early goal-directed therapy using adequate antibiotics such as IV meropenem, and source control by draining the bilateral empyema with chest tubes is warranted [17]. ARDS resolved along with the septic shock, and the patient eventually improved clinically. The role of anticoagulation is still controversial; according to anecdotes, it can prevent the formation of new septic emboli in the internal jugular veins; however, it is usually indicated only in patients with pelvic thrombophlebitis [1, 10]. In patients with uncontrolled sepsis or septic emboli despite adequate antibiotic and anticoagulation, surgical ligation or excision of the internal jugular vein might be indicated [1, 10, 18]. Ligation or resection of the IJV was frequently performed before the advent of antibiotics, and in these days, it is reserved for cases of uncontrolled sepsis with or without ongoing septic emboli despite antibiotics [2, 19]. In conclusion, a high index of suspicion should be placed for Lemierre's syndrome when a previously healthy adult presents with oropharyngeal infection and then exhibits symptoms and signs of internal jugular vein thrombophlebitis [20]. Blood cultures and appropriate imaging should provide a definitive diagnosis.

## Additional Points

*Learning Points*. (1) A high index of suspicion should be placed for Lemierre's syndrome when a previously healthy adult presents with oropharyngeal infection and then exhibits symptoms and signs of sepsis even in the absence of internal jugular vein thrombophlebitis as in our case. (2) Lemierre's syndrome can complicate to cause acute respiratory distress syndrome (ARDS). (3) *Fusobacterium* is sensitive to most of the antibiotics; however, it can be resistant to macrolides.
